# Rapid Changes in Movement Representations during Human Reaching Could Be Preserved in Memory for at Least 850 ms

**DOI:** 10.1523/ENEURO.0266-20.2020

**Published:** 2020-11-16

**Authors:** James Mathew, Philippe Lefevre, Frederic Crevecoeur

**Affiliations:** 1 Institute of Communication Technology, Electronics and Applied Mathematics, Universite Catholique de Louvain, Louvain-la-neuve 1348, Belgium; 2 Institute of Neuroscience, Universite Catholique de Louvain, Brussels 1200, Belgium

**Keywords:** adaptive feedback control, electromyogram, forcefield adaptation, motor adaptation, online corrections, via-point reaching control

## Abstract

Humans adapt to mechanical perturbations such as forcefields (FFs) during reaching within tens of trials. However, recent findings suggested that this adaptation may start within one single trial, i.e., online corrective movements can become tuned to the unanticipated perturbations within a trial. This was highlighted in previous works with a reaching experiment in which participants had to stop at a via-point (VP) located between the start and the goal. An FF was applied during the first and second parts of the movement and then occasionally unexpectedly switched off at the VP during catch trials. The results showed an after-effect during the second part of the movement when participants exited the VP. This behavioral result was interpreted as a standard after-effect, but it remained unclear how it was related to conventional trial-by-trial learning. The current study aimed to investigate how long do such changes in movement representations last in memory. For this, we have studied the same reaching task with VP in two situations: one with very short residing time in the VP and the second with an imposed minimum 500 ms dwell time in the VP. In both situations, during the unexpected absence of the FF after VP, after-effects were observed. This suggests that online corrections to the internal representation of reach dynamics can be preserved in memory for around 850 ms of resting time on average. Therefore, rapid changes occurring within movements can thus be preserved in memory long enough to influence trial-by-trial motor adaptation.

## Significance Statement

Recent studies suggested that adaptive feedback control happens within a reach movement, and the feedback responses are tuned specifically to the single-trial perturbation. Here, we show that these feedback-mediated changes in movement representations can last for around 850 ms and are available to reproduce the characteristics of the newly acquired correction process. Current data replicate previous studies showing that feedback corrections are associated with changes in online representations and demonstrate that these changes are preserved in memory long enough to be an important component of standard trial-by-trial adaptation.

## Introduction

Humans adapt to forcefield (FF) perturbation during reaching movements within a few minutes of practice (Shadmehr and Mussa-Ivaldi, 1994; Brashers-Krug et al., 1996; Shadmehr and Brashers-Krug, 1997). But learning is hampered when attempting to adapt to opposing FFs sequentially or intermittently (Gandolfo et al., 1996; Karniel and Mussa-Ivaldi, 2002; Caithness et al., 2004). The reasoning for the relative inability to learn opposing perturbations is that, given no explicit contextual information about the forcefield (FF), the motor memory attempts to learn the mean of the FFs applied on the recent trials (Scheidt et al., 2001) using a single internal model of the mean of the random environment (Takahashi et al., 2001), or the internal models for different perturbations share common resources (Tong and Flanagan, 2003). In these scenarios, it was demonstrated that the presence of explicit contextual cues associated with each perturbation or different representations could facilitate the adaptation to opposing perturbations, by acquiring multiple internal models simultaneously and predictively switching between them (Wada et al., 2003; Osu et al., 2004; Imamizu et al., 2007; Cothros et al., 2008; Addou et al., 2011; Hirashima and Nozaki, 2012).

However, more recent studies highlighted the possibility of concomitant learning of opposing and unexpected FFs that could be explained as the expression of online, continuous adaptive control (Crevecoeur et al., 2020a,b). This supports the possibility that online feedback corrections happening within a trial are not stereotyped but are associated with specific changes in movement representation (Joiner et al., 2017). It remains to be elucidated whether the underlying mechanism associated with feedback adaptation plays a role in the trial-by-trial adaptation that characterizes learning across trials.

A central piece of evidence for rapid adaptation is based on the presence of after-effects expressed after a stop-over at a via-point (VP; Crevecoeur et al., 2020b). On catch trials, an FF was applied during the first part of the movement and then unexpectedly switched off after the VP. When participants exited the VP, after-effects were observed, which were specific to the perturbation experienced before VP within the same trial and were consistent with the after-effects observed in conventional trial-by-trial adaptation scenarios. The movement after VP was interpreted as an after effect, showing that the feedback correction elicited before the VP could change movement representations online.

It remained unclear whether these changes in reach representation observed in trials with VP were short-lived or whether they could participate in trial-by-trial adaptation. More precisely, it is unknown whether the motor system forgets the effect of online feedback corrections very fast, as it arises because of transient disturbances, or if these changes are retained in memory for enough time to impact behavior in the next trial. If the online feedback corrections are retained, then it suggests that much of trial-by-trial adaptation and after-effects may be acquired within perturbed movements. Alternatively, if feedback-related changes in movement representation decay very fast, then trial-by-trial learning must be based on offline adjustments. In the present work, we replicated previous findings and found that these online feedback corrections elicited after-effects that could be retained in memory for at least 850 ms. Therefore, feedback adaptation constitutes a candidate component of more conventional trial-by-trial learning.

## Materials and Methods

### Experimental design

A total of 18 right-handed healthy adults (age = 22.9 ± 1.9, 10 female) were recruited for the study. All of them provided written informed consent. The experimental paradigm was approved by the Ethics Committee of the host institution and complied with the Declaration of Helsinki. Participants were compensated for their participation.

Participants grasped the handle of a robotic arm (KINARM) and were instructed to perform visually guided forward reaching movements toward a virtual target and their forehead resting on a stable resting pad, to minimize head movements.

**Figure 1.**
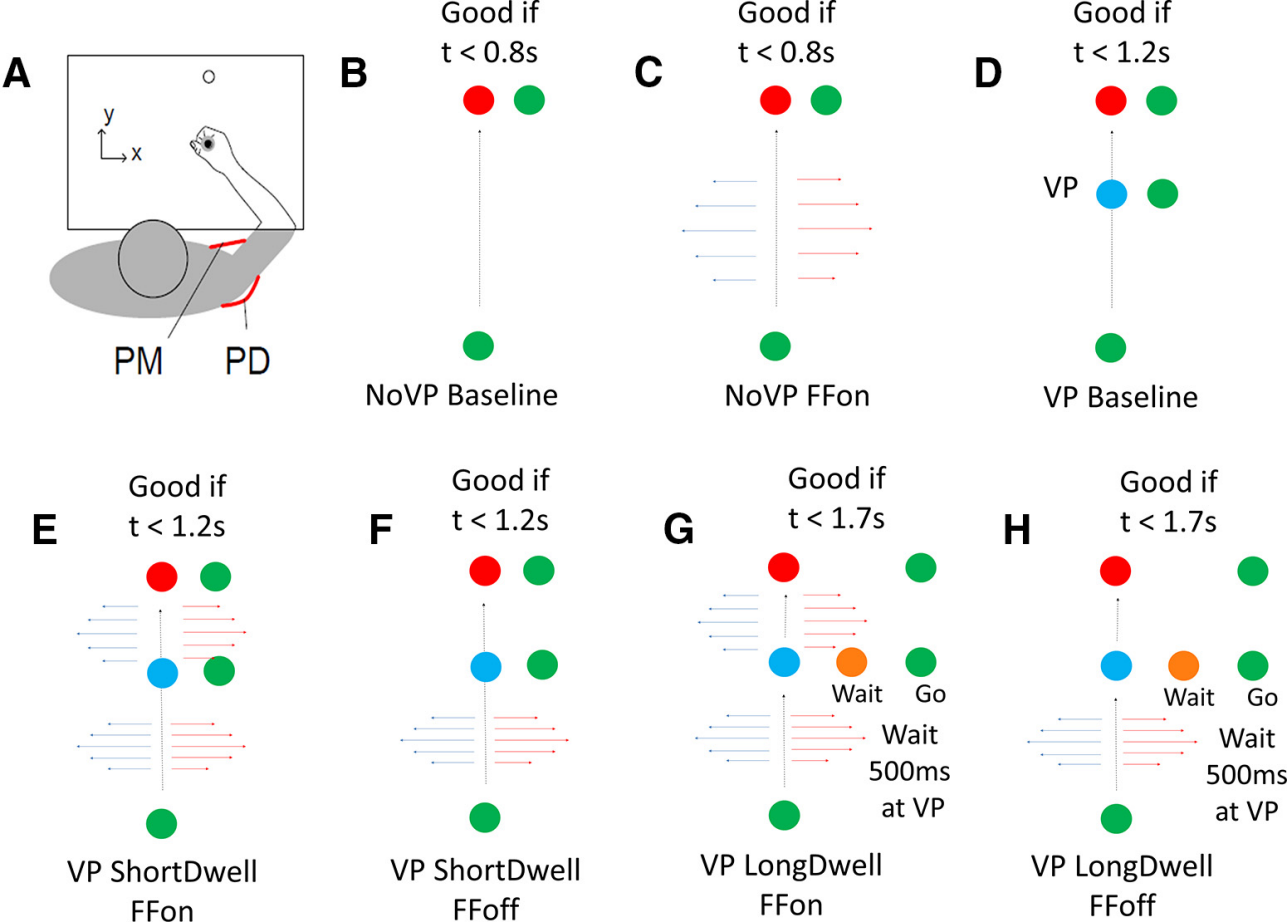
***A***, Experimental setup. Electromyogram was recorded from PM and PD. ***B***, Typical simple reaching trial without VP and FF. ***C***, NoVP trial with FF, either CW (red arrows) or CCW (blue arrows). ***D***, VP trial without FF. Participants were requested to make a short pause at VP and proceed to the final target. ***E***, VP ShortDwell trial with FF before and after VP. ***F***, VP ShortDwell catch trial with FF unexpectedly turned off after VP. ***G***, VP LongDwell trial with FF before and after VP. ***H***, VP LongDwell catch trial with FF unexpectedly turned off after VP. The target would change color when the participants made movements within the instructed time window. For ***D–H***, VP would change color when the participants made a pause at VP and the hand velocity dropped to <3 cm/s. For ***G***, ***H***, VP would change color again when the participant stayed at VP for 500 ms.

There were mainly seven types of movement conditions:

(1) No VP trials (NoVP baseline/B). Participants had to wait at the starting position (a filled circle with radius 0.6 cm) for a random delay uniformly distributed between 2 and 4 s (Fig. 1*B*). The goal position was fixed at 15 cm from the starting point and was initially presented as an open red circle. A cue was delivered to initiate the movement by filling the circle in the goal position and for a successful trial, the participants had to reach the goal position within 600–800 ms (including reaction time) and stabilize there for at least 1 s. Visual feedback was provided to inform them about the reaching time. If they moved too fast, the goal circle changed back to an open circle. If the movement was too slow, it remained red. The goal target became green when they hit it within the specified time window. When they managed to keep the cursor in the goal target for the instructed stabilization period, the trial was successful, and a score displayed on the screen was incremented (one point). The scores and feedback about timing were provided to encourage consistent movement times, but all trials were included in the dataset. In all cases, the direct vision of the arm and hand was blocked but the cursor aligned to the handle was always visible.

(2) NoVP with FFs (NoVPFF). In these types of trials, participants experienced mechanical perturbation by orthogonal FFs during the forward movement, i.e., lateral force proportional to the forward hand velocity (F_x_ = ±L$v_{y}$, L = 13 Nsm^−1^). FFs were either clockwise (CW) or counter-CW (CCW) (Fig. 1*C*).

(3) VP baseline trials with slowdown at VP. In these trials, a VP (filled blue circle of radius 1 cm) was located at 10 cm on the straight line joining the start and goal position. Participants were instructed to reach the goal position through the VP (Fig. 1*D*). Bonus points (three points) were given when they paused at the VP and the hand velocity inside the VP dropped below 3 cm/s. Feedback about a successful slowdown at the VP was given during the trial by changing the color of the VP filled circle from blue to green.

(4, 5) VP trials with short dwell time at VP, FF ON before VP, and ON/OFF after VP (VPsFFon/VPsFFoff). For catch trials, the hand velocity was monitored online so as to turn off the FF if the hand cursor was at the VP and the hand velocity dropped below 3 cm/s while at the VP (as shown in Fig. 1*E,F*).

(6, 7) Same as 4 and 5, but with long dwell time at VP (VPlFFon/VPlFFoff). In these trials, participants were forced to stay at the VP for at least 500 ms before they proceed to the goal position. Once they reached the VP, the filled blue circle became orange, and after 500 ms, it turned to green, which was a go cue to proceed to the final position (Fig. 1*G,H*). The notation VPs/lFFon and VPs/lFFoff corresponds to VP trials with FF on and off in either ShortDwell or LongDwell situations. To summarize, we had a factorial design of trials: No VP trials with FF on or off (CW/CCW); VP trials with FF on or off, catch trials where the FF was turned off after the VP, and similar VP trials with the instruction to remain in the VP for at least 500 ms (CW/CCW, ShortDwell/LongDwell, FF ON/OFF after VP).

Participants performed two different kind of blocks: ShortDwell and LongDwell. Each consisted of 240 trials composed of 100 NoVP baseline trials, 40 NoVPFF trials, 20 VP baseline trials, 40 VPs/lFFon, and 40 VPs/lFFoff trials. All trials and FF directions were interleaved randomly within each block, such that the occurrence and direction of the perturbations were unpredictable. Half of the participants did ShortDwell block set before LongDwell block set.

### Data analysis and statistics

The two-dimensional coordinate of the cursor aligned to the robotic handle, and the forces at the interface between the participants’ hand and the handle were sampled at 1 kHz. Signals were digitally low-pass filtered with a fourth-order, dual-pass Butterworth filter with a cutoff frequency of 50 Hz. Velocity signals were obtained from numerical differentiation of position signals (fourth order, finite difference algorithm). Electromyogram (EMG) was recorded from the shoulder flexor, pectoralis major (PM), and shoulder extensor, posterior deltoid (PD), the main muscles recruited when performing lateral corrections against the perturbations used in this experiment (Bagnoli Desktop System, Delsys). EMG electrodes were positioned over the muscle belly after light abrasion of the skin. A dermatrode self-adhering electrode was positioned on the right foot ankle for ground. EMG signals were collected at 1000 Hz sampling frequency and digitally bandpass filtered (fourth order dual-pass: [10, 400] Hz).

Three events were used as timing references. First, reach onset was defined as the moment when the cursor exited the home target. Second, the moment the cursor reached VP. Third, the moment the cursor exited VP and proceeded toward the final target location. Hand paths were averaged first, within and then across the subjects. For each subject, lateral ($v_{x}$) and forward ($v_{y}$) component of the peak velocity was computed before and after VP for each trial and averaged per condition. EMG traces were averaged offline, first within and then across subjects and were aligned to the VP exit to compute the characteristics of muscle response after VP, in both ShortDwell and LongDwell, VPs/lFFoff and VPs/lFFon. For statistical comparison across conditions, EMG was averaged in 100 ms bins before VP exit. Paired *t* tests and repeated-measures ANOVAs were used to find a significant difference with *p* < 0.05. We measured the onset of changes in EMG after the VP exit. EMG traces averaged across trials for each subject were collapsed into a 30 ms wide (centered) sliding window, and sliding comparisons through time were performed with paired *t* tests. We searched in the time series of *p* values the moment the difference across populations of EMG data crossed a threshold (*p* < 0.05), which was immediately followed by a strongly significant difference (*p* < 0.001).

## Results

### Behavioral traces for simple movements

**Figure 2.**
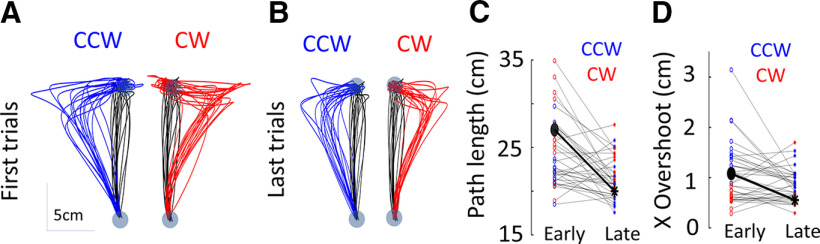
***A***, First trials of no VP movements with CCW (blue) and CW (red) FFs (NoVPFF) plotted with baseline (NoVP) trials (black) for all 18 subjects. ***B***, Last trials of the same, note how the target overshoot is reduced in comparison with first trials. ***C***, Distance traveled (path length) by hand during first and last trials. ***D***, End-point target overshoot in the X direction in early and late trials. The thick black dot and star in ***C***, ***D*** indicate the mean. There is a significant reduction in target overshoot and path length between early and late trials, which show hints of adaptation.

Simple reaching trials with and without perturbation and adaptation across trials were discussed in detail in some previous works (Crevecoeur et al., 2020a,b). As in these previous reports, for no VP trials, the current dataset also observed a significant reduction in end-point target overshoot (*t* test: *t*_(35)_ = 3.77; *p* < 0.001) and hand path length (*t* test: *t*_(35)_ = 4.33; *p* < 0.001) between the first and last trials (Fig. 2), suggesting that feedback correction for the unanticipated FF trials improved.

### VP dwell time

**Figure 3.**
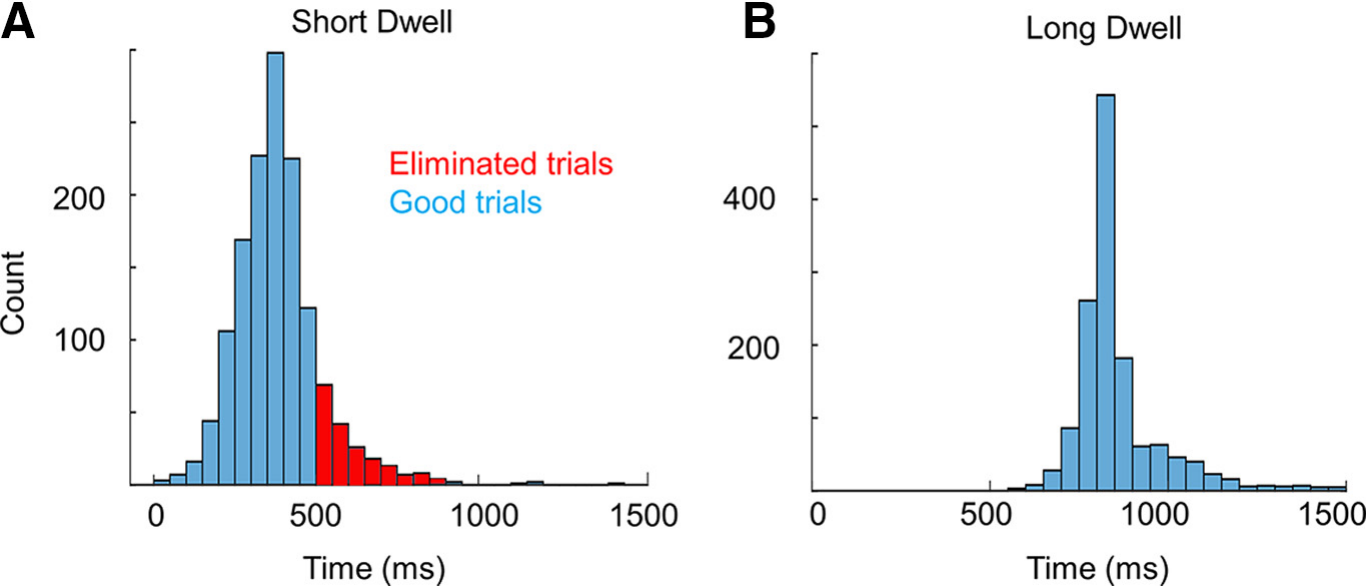
Distribution of actual dwell times for all subjects and all trials in (***A***) ShortDwell and (***B***) LongDwell blocks. Each bin corresponds to 50 ms. For ShortDwell, the trials with dwell times longer than 500 ms and for LongDwell, the trials with dwell times shorter than 500 ms or longer than 1500 ms were removed from the analyses.

Turning to VP trials, we measured the actual dwell time for short and long dwell conditions to verify whether participants complied with task instructions (Fig. 3). For analysis, we defined dwell time as the time interval between the VPentry and VPexit for the hand motion. In ShortDwell trials, the mean dwell duration at VP was 345 ± 22 ms (for all participants, min = 22 ms; max = 499 ms). Around 15% of the total ShortDwell trials (variation per subject: 0–37.5%) consisted of dwell duration not in the range 0–500 ms were eliminated from further analysis (Fig. 3*A*, red fraction of the histogram). In LongDwell, mean dwell duration was 856 ± 37 ms (min = 550 ms, max = 1491 ms). In this condition, 3% of the total LongDwell trials from all the subjects (variation per subject: 1.25–6.25%) were eliminated, so that LongDwell trials consisted of dwell time within the range 500–1500 ms (Fig. 3*B*).

### Behavioral traces for movement through VP

To trace out the nature of online changes in movement representation happening within the trial, we compared the movement after VP in situations with FF on or off after the VP (VPs/lFFon and VPs/lFFoff).

#### FF off (catch trials)

**Figure 4.**
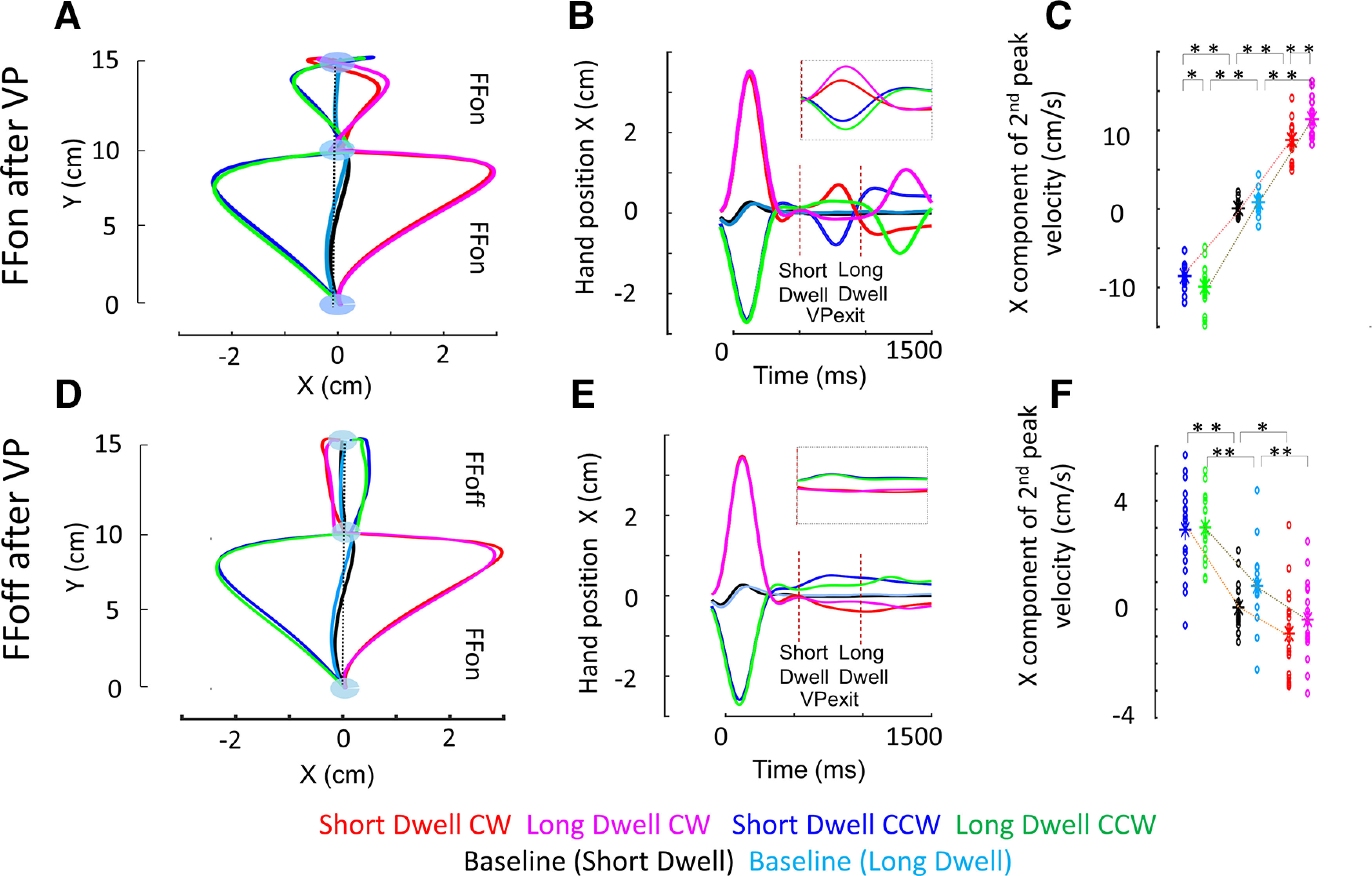
***A–C***, Condition where FF was on before and after VP for both CW and CCW perturbations. ***D–F***, Condition where FF was on before VP, but turned off after VP. VP baseline trials were included for comparison. ***A***, ***D***, Mean hand position in space. The dotted vertical line represents zero deviation. ***B***, ***E***, Mean X hand position across time from the onset of reach. The dotted lines in brown represent the mean VP exit time points. The plots in the dotted inset box (*y*-axis = [−2 2], *x*-axis = [0 600]) represent the mean X hand position immediately after VP exit for the next 600 ms. ***C***, ***F***, Lateral component of the maximum hand velocity after the VP; **p < 0.001, *p < 0.01.

VPs/lFFoff trials were interleaved as catch trials to capture the dynamics of online correction within the trial. When the FF was off after VP, an after-effect was observed in the subsequent chunk of movement, such that the hand path deviated on the other side compared with that of the previously experienced perturbation before VP (Fig. 4*D*,*E*). The lateral component of the second peak velocity showed inverse modulation in comparison to the hand path deviation experienced before the VP (Fig. 4*F*). There was a significant difference from baseline trials for CCW and CW in ShortDwell (CCW: *t*_(17)_ = −8.85; *p* < 0.001 and CW: *t*_(17)_ = 3.03; *p* < 0.01) and LongDwell (CCW: *t*_(17)_ = −8.89; *p* < 0.001 and CW: *t*_(17)_ = 5.80; *p* < 0.001). But there was no significant difference between ShortDwell and LongDwell trials for CCW (*t*_(17)_ = −0.25; *p* = 0.80) and CW (*t*_(17)_ = −1.43; *p* = 0.17). This showed that the dynamics of the online correction adopted before VP were continued after VP and it was comparable for ShortDwell and LongDwell conditions. That means this correction strategy was preserved and exploited even after a pause of around 850 ms at the VP.

One possibility was that participants performed faster movements after the VP in the LongDwell condition, resulting in larger forces produced by the FF, but it was not the case: we also checked the Y component of the second peak velocity in VPs/lFFoff trials and found no significant difference from baseline trials for CCW and CW in ShortDwell (CCW: *t*_(17)_ = −1.72; *p* = 0.10 and CW: *t*_(17)_ = −1.74; *p* = 0.1), but there was a significant difference in LongDwell (CCW: *t*_(17)_ = 4.02; *p* < 0.001 and CW: *t*_(17)_ = 5.89; *p* < 0.001). Also, there was no significant difference in the Y component between ShortDwell and LongDwell trials for CCW (*t*_(17)_ = 1.13; *p* = 0.27), but there was for CW(*t*_(17)_ = 2.87; *p* = 0.01).

In addition, we have done a separate analysis by further splitting the LongDwell trials according to the individual median dwell time (range: 732–867 ms; mean = 821 ms), so that Early-LongDwell was within the range 500–821 ms (mean = 773 ms) and Late-LongDwell was within the range 821–1500 ms (mean = 934 ms). In this case also, when FF was off after VP, there was no significant difference between ShortDwell and Early-LongDwell (CCW: *t*_(17)_ = −0.89; *p* = 0.38 and CW: *t*_(17)_ = −1.63; *p* = 0.12), as well as ShortDwell and Late-LongDwell (CCW: *t*_(17)_ = 0.67; *p* = 0.51 and CW: *t*_(17)_ = −0.46; *p* = 0.65). Thus, the consistent after-effects were observed regardless of the average duration between the two consecutive chunks of movement in the range 0–1500 ms (Fig. 3).

#### FF on

When the FF was on after VP, participants made lateral deviation (which was evident in Fig. 4*A*,*B*) as expected and the modulation of lateral (x) component of the second peak velocity shows a similar trend as that of the perturbation they have experienced before VP (Fig. 4*C*). Simple *t* tests on the x component of second peak velocity showed a significant difference from baseline trials for CCW and CW in both ShortDwell (CCW: *t*_(17)_ = 23.34; *p* < 0.001 and CW: *t*_(17)_ = −17.42; *p* < 0.001) and LongDwell (CCW: *t*_(17)_ = 20.22; *p* < 0.001 and CW: *t*_(17)_ = −16.65; *p* < 0.001) situations. This was somehow expected as the duration of pause at VP may not directly influence the peak velocity modulation trend for subsequent perturbation, in comparison with baseline trials. Also, there was a significant difference in the x component between ShortDwell and LongDwell trials for CCW (*t*_(17)_ = 2.41; *p* = 0.03) and CW(*t*_(17)_ = −4.86; *p* < 0.001).

We checked the Y component of the second peak velocity in VPs/lFFon trials, and there was no significant difference from baseline trials for CCW and CW in ShortDwell (CCW: *t*_(17)_ = 1.59; *p* = 0.13 and CW: *t*_(17)_ = −0.99; *p* = 0.34), but significant difference in LongDwell (CCW: *t*_(17)_ = 5.56; *p* < 0.001 and CW: *t*_(17)_ = 5.20; *p* < 0.001). In addition, there was no significant difference in the Y component between ShortDwell and LongDwell trials for CCW (*t*_(17)_ = 1.62.; *p* = 0.12) and CW(*t*_(17)_ = 1.68; *p* = 0.11). Thus, participants control was more sensitive to the FF after the VP in the long-dwell condition. The analysis of EMG below supports this explanation.

### EMG activity

**Figure 5.**
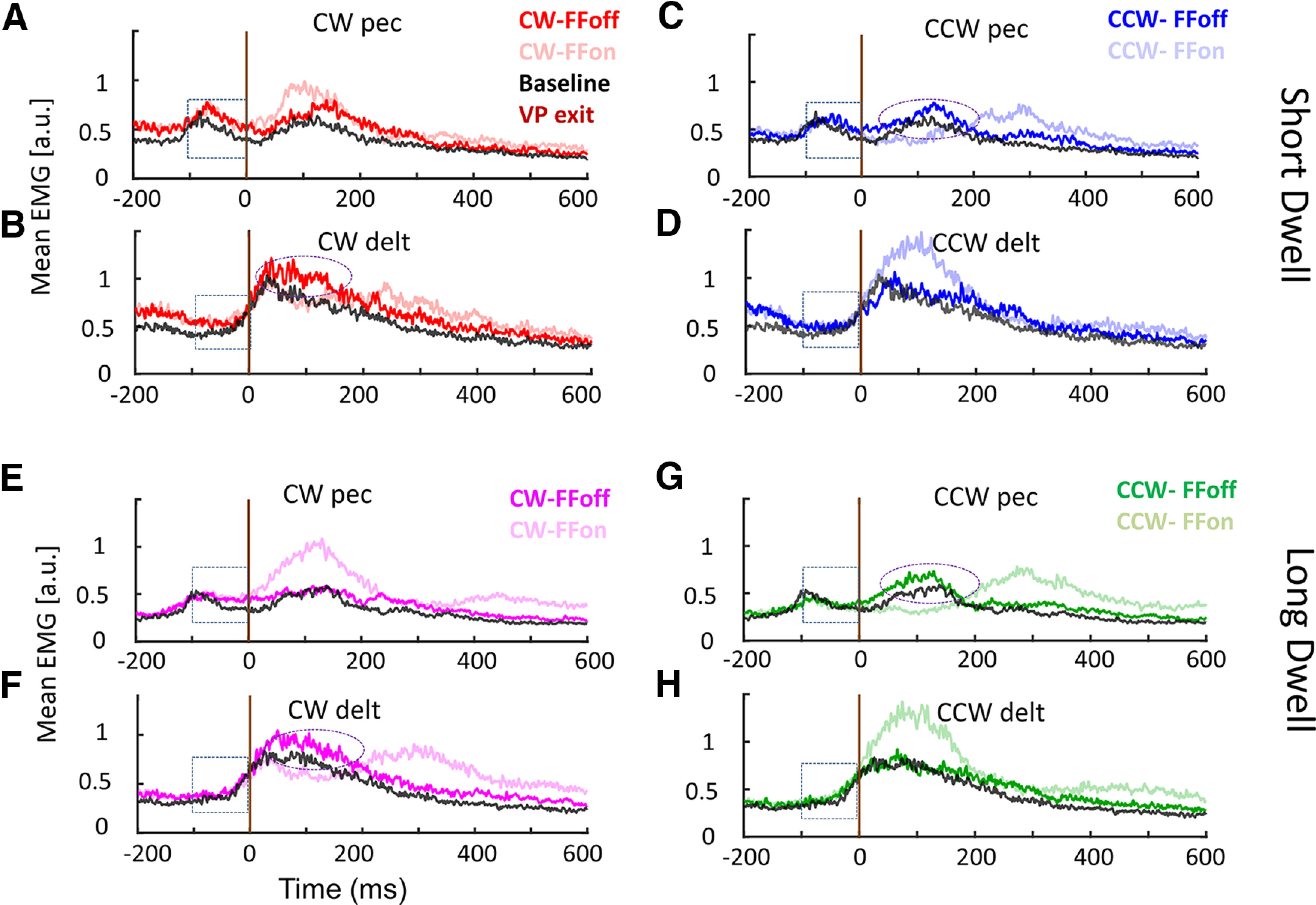
EMG correlates after VP in ShortDwell (***A***, ***B***, ***C***, ***D***) and LongDwell (***E***, ***F***, ***G***, ***H***) trials. Each trace represents mean across subjects. ***A***, ***B***, ***E***, ***F***, CW perturbation, ***C***, ***D***, ***G***, ***H***, CCW perturbation. All EMG traces were aligned at the time of the VP exit (dark brown line), the “0 ms” in the horizontal axis indicates the timing of the VP exit. FF on (VPs/lFFon, light shade) and off conditions (VPs/lFFoff, dark shade) were compared with VP baseline trials. The dotted oval shape represented higher activation in antagonist muscle; 100-ms time window before VP exit was highlighted in dotted square boxes.

Previous studies showed that unexpected perturbation elicits an increase in coactivation (Milner and Franklin, 2005; Franklin et al., 2008; Crevecoeur et al., 2019) to counter following disturbances. It remains debated whether co-contraction increases the intrinsic stiffness of muscles, making the joints mechanically rigid (Burdet et al., 2001; Gribble et al., 2003) or whether the advantage of co-contraction is to increase feedback gains and make neural control more robust (Pruszynski et al., 2009; Crevecoeur and Scott, 2014; Crevecoeur et al., 2019). Importantly, we found in the current dataset that an increase in coactivation was also elicited within movements including a stopover at the VP, and the comparison of trials with FF on after the VP allowed us quantifying the effect of co-contraction on behavior. We investigated EMG levels of an antagonist pair of shoulder muscles as well as correlates of after-effects across the two conditions. We plotted EMG traces after VP in VPs/lFFon and VPs/lFFoff situations for both PM and PD (Fig. 5).

To evaluate whether there was any difference in muscle activity at VP exit across ShortDwell and LongDwell, we computed the difference of mean EMG activity in a window 0–100 ms before VP exit, between the two dwell conditions and performed two-way repeated-measures ANOVA over factors: muscle (MUS: PEC vs DELT) and FF (on vs off) and found there was no effect of MUS (*F*_(1,143)_ = 2.77; *p* = 0.11), FF (*F*_(1,143)_ = 1.12; *p* = 0.30), and their interaction (*F*_(1,143)_ = 3.50; *p* = 0.08). There was no significant difference between FF on/off situations in agonist and antagonist muscles. In other words, in both FFon and FFoff conditions (ShortDwell vs LongDwell), we measured similar correlates of agonist-antagonist activity just before the exiting the VP, that accounted for the after-effects when the FF was unexpectedly turned off.

We also calculated the mean EMG activity 100 ms before the VP exit (Fig. 5, dotted square boxes) and the difference between baseline and ShortDwell (CW/CCW/FFon/FFoff pooled together, PEC: *t*_(71)_ = −8.24; *p* < 0.001 and DELT: *t*_(71)_ = −6.19; *p* < 0.001) as well as baseline and LongDwell (PEC: *t*_(71)_ = −2.6; *p* = 0.01 and DELT: *t*_(71)_ = −5.9; *p* < 0.001). The higher muscle activity in both muscles compared with baseline trials could raise the possibility of co-contraction just before the VP exit.

Then we calculated the pairwise difference between ShortDwell and LongDwell trials for each subject for VPs/lFFon and VPs/lFFoff situations for each muscle. For FF on condition, the overall muscle activity in a window 0–100 ms before VP exit was less in amplitude for LongDwell compared with ShortDwell (FF on, CW/CCW pooled together; PEC: *t*_(35)_ = −5.35; *p* < 0.001 and DELT: *t*_(35)_ = −4.78; *p* < 0.001). That means there was a tendency to co-contract at the VP, which decreased over the course of residing time in the VP. Importantly, for FF off condition also, the overall muscle activity 0–100 ms before VP exit was less in amplitude for LongDwell compared with ShortDwell (FF off, CW/CCW pooled together; PEC: *t*_(35)_ = −5.35; *p* < 0.001 and DELT: *t*_(35)_ = −5.25; *p* < 0.001), which allows us to confirm that it had no influence on the after effect. In principle, co-contraction would result in a trajectory that is less sensitive to perturbation or errors, thus these results were expected. The foregoing analysis provides a confirmation: we observed different levels of co-contraction while similar after-effect, suggesting that the level of coactivation was not statistically related to the after-effect. Interestingly we observed that the trajectories after the VP were more impacted in the LongDwell condition when the FF remained on, which likely resulted from a combination of changes in representation (highlighted by the after-effects) and from a change in control gains associated with co-contraction.

We further sought to characterize EMG correlates of FF anticipation after the VP by performing comparisons based on a sliding window. EMG agonist (CW pec, CCW delt) followed a similar profile with and without FF until after VP, and this activity was higher than baseline. The antagonist (CCW pec, CW delt) muscle modulation also reflected correction for the unexpected deviation induced by switching off the FF (Fig. 5, dotted oval). A similar observation was made in LongDwell trials. We have conducted paired *t* tests for the mean of 30-ms sliding window for the FFon and FFoff conditions and found the time points at which the on/off traces started to show a significant difference from VP exit, for ShortDwell: agonist pair = −22 ± 15 ms, antagonist pair = −14 ± 15 ms and for LongDwell: agonist pair: = −26 ± 15 ms, antagonist pair: = −37 ± 15 ms. (The average hand tangential velocity at the exact moment of VPexit was 0.23 ± 0.03 cm/s for ShortDwell and 0.24 ± 0.02 cm/s for LongDwell.)

To summarize the analyses of EMG results, we highlighted a spontaneous tendency to co-contract at the VP, which decayed over the course of the dwell time interval. This tendency had an impact on the perturbation-related motion when the FF remained on likely because of intrinsic changes in limb impedance and to an increase in robustness. In contrast, there was no systematic directional bias in muscle state that could account for the rapid after-effect in either condition (short and long dwell times), and these after-effects were statistically similar despite the difference in co-contraction. In addition, we reported correlates of agonist activity when exiting the VP, and antagonist response when the FF was turned off. These detailed EMG analyses allowed us to emphasize the impact of co-contraction, and to dissociate it from the near-instantaneous after-effects that, we found, could be preserved in the motor system for at least 850 ms.

## Discussion

We investigated the temporal nature of rapid changes in movement representation because of unexpected FF perturbations. For this, we have studied the reaching task through a VP on the pathway in two situations: one with very short residing time at the VP (ShortDwell) and the second with an imposed minimum 500 ms dwell time at the VP (LongDwell). First, in support of previous works (Crevecoeur et al., 2020a,b), we observed feedback adaptation across no VP trials evidenced by a reduction in target overshoot and path length, when opposing FFs were applied randomly. That means online feedback corrections were tuned to specific perturbation within each individual trial although the FFs could not be anticipated. Additionally, the current dataset replicated the previous findings (Crevecoeur et al., 2020a) that EMG imprints of changes in feedback corrections occurred within 250 ms of reach onset, with this cohort of participants (data not shown). Second, in VP trials, we observed the presence of an after-effect to the movement correction after the VP that was opposed to the perturbation experienced before, when participants exited the VP in <500 ms (ShortDwell). Similar to standard after-effects evoked by a single FF trial, the presence of after-effects after the VP was understood as the expression of changes in movement representation occurring online. Third, comparable after-effects in terms of hand path deviation and peak lateral velocity were observed in behavior and EMG recordings with an average 850-ms dwell time at VP (LongDwell) and even beyond as observed when the trials were split according to their actual dwell time. In all, our results showed that somatosensory feedback about movement error could have long-lasting effects and be preserved during intervals comparable to different conditions of movement planning as in the context of trial-by-trial adaptation.

Our main motivation was to relate rapid after-effects evoked within a sequence of movements with residing times of the order of <500 ms to those expressed in a time scale closer to a second. In standard reaching experiments, consecutive trials are typically separated by a few seconds. A direct comparison between after-effects after the VP and those following standard FF trials could not be conclusive because the limb configuration is not the same; however, since these rapid feedback-related adjustments were preserved up to a time interval closer to one second, we suggest that they play a central role in well-known standard trial-by-trial learning. In other words, trial-by-trial adaptation and after-effects would result at least partially from within-movement neural processing associated with feedback control.

### Adaptation of online feedback correction across trials

In FF adaptation experiments, one common assumption is that the prediction by forward models cannot change within a movement because of sensory delays, hence this mechanisms would only be available after the trial and therefore the reaching movements that employ only a feedforward controller could not account for the within-trial adjustments (Wada et al., 2003; Yousif and Diedrichsen, 2012). In such cases, since the motion-dependent adaptive responses cannot be based on real-time sensory feedback, the feedforward adaptive responses must be programmed in advance based on predictions (Sing et al., 2013), and the delayed feedback responses were used to learn a predictive feedforward response (Thoroughman and Shadmehr, 1999). Alternatively, real-time online corrections could be achieved through a feedback controller that must contain a forward model capable of accurate real-time prediction of the state of the limb, and combine the state predictions (Wagner and Smith, 2008). In the case of random inconsistent perturbations scenarios, it was shown that the gain of sensory feedback responses appears to increase (Liu and Todorov, 2007) and feedforward adaptive responses to decrease (Gonzalez Castro et al., 2014). Such cases highlighted “the adaptation of online feedback correction” (Yousif and Diedrichsen, 2012; Joiner et al., 2017), more specifically “feedback adaptation,” i.e., trial-by-trial fine-tuning of feedback responses to the specific perturbation happening within each trial (Crevecoeur et al., 2020b). In our case, across trials, there is an accumulation of learning, but this is not because of the predictive factor, instead, we argue that this is because of the feedback-mediated online corrective process (feedback adaptation) since it is expressed very quickly in the ShortDwell condition.

In line with these concepts, it was demonstrated that the prediction of the current state of the limb could happen within long-latency feedback pathways (latency ∼60–100 ms; Crevecoeur and Scott, 2013; Scott, 2016; Crevecoeur and Kurtzer, 2018), which is faster than trial time. Long-latency feedback correction is also sensitive to FF adaptation and can facilitate trial-by-trial learning (Ahmadi-Pajouh et al., 2012; Cluff and Scott, 2013; Maeda et al., 2020). Thus, this pathway may support sensory-prediction and adaptation functionally, and its latency relative to reach time leaves time for potential changes within a movement. Without restricting to long-latency feedback, changes in feedback response were measured within ∼250 ms of reach onset (Crevecoeur et al., 2020a). In all, we believe that the assumption that movement representations are fixed within a reaching movement requires revision.

### Traces of online feedback correction as after-effects in VP trials

In case of any perturbation during reaching movement, within a short period of movement initiation, sensory feedback starts to influence motor command updating throughout the movement (Lackner and Dizio, 1994; Brashers-Krug et al., 1996; Kawato, 1999; Shadmehr et al., 2010; Wolpert et al., 2011). Our developments suggest that a change in movement representation occurs in parallel during movement. Then the question is how long does this movement representation persist in the memory? Conventional trial-by-trial studies reported the variation in hand dynamics and movement trajectory from one trial to another.In these situations, there was an intertrial interval of 1 or 2 s to return the hand to the starting position either passively or actively (Gandolfo et al., 1996; Donchin et al., 2003; Tong and Flanagan, 2003; Caithness et al., 2004; Smith et al., 2006; Kording et al., 2007; Izawa et al., 2008). The idea of providing VPs in between the start and final position facilitated tracking the online feedback-mediated corrective processes and associated adaptation within chunks of movement.

In our case, first, during ShortDwell condition, we have observed clear after-effects during the follow-through movement after VP (Fig. 4*D–F*). Why did the hand path deviate after VP? After-effects are known to reveal the change in motor command and the nature of the adaptive process by highlighting the discrepancy between expected and actual dynamics (Shadmehr and Mussa-Ivaldi, 1994; Bhushan and Shadmehr, 1999). During the first phase of the movement, the hand experienced perturbation and it has been shown that the hand force to counteract this disturbance became tuned to the hand velocity, which was consistent with online adaptation (Crevecoeur et al., 2020b). This corrective process and thus evolved movement representation continued after VP as the CNS expected a continuation of perturbation. In the absence of this, the discrepancy in the expected and actual hand dynamics resulted in an after-effect.

### Long-lasting effects of somatosensory feedback about movement error: a basis of trial-by-trial adaptation?

Rapid feedback adaptation may still differ from the mechanism engaged in memory retrieval associated with different planning conditions. Indeed, follow-through movements with a short movement pause at VP reported that sensorimotor states that differ in their recent temporal history (∼600 ms) could engage distinct representations, but importantly they decayed over time (Howard et al., 2012). That means, the more you rest at VP (or stay at intertrial interval period), the associated plan started to fade and the memory of the FF could not be recalled; Wainscott and colleagues reported a reduction in the interference of random perturbation learning, when a perturbed movement followed the distinct previous movement, within a time interval of 500 ms (Wainscott et al., 2005). Also, even contextual premovement (Sarwary et al., 2015), planning (Howard et al., 2015; Sheahan et al., 2016), or motor imagery (Sheahan et al., 2018) of the second phase of follow-through movement could facilitate simultaneous learning of opposing perturbations, provided with a short dwell time at a VP.

Our results contrast with differences in planning associated with a VP, since in our case we still observed significant after-effects during the second phase of the movement (Fig. 4*D–F*), after residing time that exceeded the time associated with a decay in the motor memory of 600 ms. In light of these previous observations, the fact that the after-effects evoked within a sequence with a VP are stable across residing time intervals, is not a trivial result. Indeed, one could have expected that the lifetime of the rapid adjustment paralleled its time scale, as proposed in previous models that consider multiple time scales of adaptation (Kording et al., 2007). In this scenario, it was reasonable to expect that a feedback related change in movement representation within ∼400 ms (from starting point to VP), would disappear within the next time window of similar length. It was clearly not the case. Thus, it seems that feedback-related changes in representation and differences evoked by distinct planning conditions do not have the same dynamics and their respective roles in trial-by-trial adaptation remain to be elucidated.

In our case, the feedback correction before the VP could play the same role as the lead-in movement, but surprisingly, we did not observe that it decayed so quickly, and our data suggested that it could even carry over across trials. The presence of feedback correction even after a short pause of around 0 to 1500 ms time duration at VP suggested that somatosensory feedback about movement error could have long-lasting effects in comparison with different conditions of planning, and these effects might constitute the basis of standard trial-by-trial learning.

Our results support the view that standard aftereffects may directly follow from feedback adjustments instead of requiring a subsequent re-planning. In ShortDwell scenario, we found that the aftereffects are the result of online feedback control, rather than feedforward control in the classical sense as this task did not involve a re-planning separated in time from the first segment, instead, the aftereffect must have been related to immediate feedback adjustments before the VP. Similar aftereffects are observed in LongDwell condition, which means that feedback-related change in movement representation is preserved and used during the second chunk of movement. We exploited LongDwell condition as a bridge between ShortDwell and ordinary trial-by-trial adaptation, so as to highlight the role of within movement neural processing associated with feedback control.

The results in the current study are in a way congruent with others who tested different intertrial intervals, such that the feedback error memory trace (which sustains up to 4 s) continuously promotes adaptation until the next movement (Huang and Shadmehr, 2007). Here, we attempted to dissociate feedback-related aftereffect that is preserved, from possible build-up or consolidation, which requires longer intertrial interval (Bock et al., 2005; Francis, 2005; Huang and Shadmehr, 2007). Reduced intertrial interval evokes error sensitive (feedback) component to play a prominent role in adaptation than predictive–error-insensitive component.

### Feedforward and feedback processes interaction

We clearly concentrate on feedback adaptation and highlight its potential contribution, but we do not reject the possibility that changes in movement representation occur offline, similar to feedforward adaptation. In fact, previous studies have suggested that the feedback response and feedforward adaptation could work independently at least to some extent (Yousif and Diedrichsen, 2012; Kasuga et al., 2015). However, our study raises the question of how much feedback and feedforward adaptation truly differ, or under which circumstances does one process prevail or influence the other. When we consider the no VP trials with FFs, intermixed with baseline and VP trials, the influence of the anticipation component was absent. In order to reveal the characteristics of online feedback corrections and feedback adaptation, we exploited VP trials. Since the movements observed as after-effects account for both feedforward force patterns and feedback control gains (Bhushan and Shadmehr, 1999), the after-effects observed in our task during the second phase of movement carry the imprint of changes in feedback responses, that might eventually update the feedforward process. In such case, it could be that gradual changes in feedforward representation result from repeated feedback corrections, which contrasts with previous models according to which changes in feedback control follow feedforward adaptation (Wagner and Smith, 2008; Ahmadi-Pajouh et al., 2012; Maeda et al., 2018). Ultimately, we do not discard feedforward control and we recognize that there can be a sequential (feedback–feedforward–feedback) update at a time scale faster than a trial but we believe it is more accurate to describe our findings in terms of an internal representation, which changes very rapidly and within a trial.

## Conclusion

In conclusion, our data further support that the fast time scales of motor adaptation are sufficiently fast to influence an on-going movement, and the associated changes in movement representation are preserved during intervals of time comparable to different conditions of planning. Hence the imprints of online feedback adaptation could be a major component of trial-by-trial adaptation in the motor system.

## Synthesis

Reviewing Editor: Leonard Maler, University of Ottawa Decisions are customarily a result of the Reviewing Editor and the peer reviewers coming together and discussing their recommendations until a consensus is reached. When revisions are invited, a fact-based synthesis statement explaining their decision and outlining what is needed to prepare a revision will be listed below. The following reviewer(s) agreed to reveal their identity: Daichi Nozaki.

Editor comments

Both reviewers agree that the research presented in this manuscript is both interesting and important. The reviewers also agree that the experiments were well designed and the data clearly supported most of the author’s conclusions.

There was agreement that the manuscript was too dense in places. I have included the main criticisms and suggestions by both reviewers. They clearly describe the fairly minor revisions that should be made to make the strong results reported here appropriate for eNeuro.

Reviewer #1

A previous study had reported that when the force-field was imposed on the 1st part of two-consecutive reaching movement going through a via-point (VP), the aftereffect was observed in the 2nd part of the movement (Crevecoeur et al., eNeuro 2020). Crevecoeur et al. assumed that the presence of aftereffect was evidence that the motor adaptation occurred online. The present study has demonstrated this adaptation effect was maintained even when increasing the dwell time at the VP to ∼850ms.

Major points:

1. The experiments were well designed, and the data were unambiguous, but I was confused with the study’s motivation. If the two-reaching movement was separated in time, the presence of aftereffect was not unexpected because the ordinary trial-by-trial adaptation took place. The authors predicted that the aftereffect would not be observed in the LongDwell condition? I might miss the critical points, but the authors need to explain more clearly why examining the adaptation for the LongDwell condition was significant.

2. I do not fully agree with the idea that the force-field adaptation occurred within the reaching movement. Nevertheless, even if the adaptation occurred offline, a possibly interesting point of the present data was that the aftereffects were not different between the Short- and LongDwell conditions. According to previous studies investigating the effect of inter-trial interval on motor adaptation (e.g., Huang & Shadmehr, J Neurophysiol 2007), a sufficient amount of time should be necessary for adaptation to evolve fully. Obviously, in the ordinary reaching paradigm, investigating the very short inter-trial interval examined in the present study was impossible. Thus, the current results were invaluable to demonstrate the unknown features of motor adaptation. Do the authors have any comments on this point? I would also like to hear the authors’ opinion that they should predict whether the aftereffect increased or not if they further increased dwell time.

Minor points:

The style of citation (e.g., F. Crevecoeur et al., Frederic Crevecoeur et al. etc) should be corrected.

Fig.5: It would be helpful to describe clearly that the “0ms” in the horizontal axis indicates the timing of the VP exit.

Line 204: The space before “x” should be removed.

Line 286: “PEC::” should be “PEC:”. “-” was duplicated in several statistics (e.g., t35=--5.35).

Reviewer #2

My main criticism is that the manuscript could be written a bit more clearly in places as it comes across as slightly ambiguous sometimes. In particular, it would be helpful to very clearly explain the task used in this and the previous work at the beginning of the manuscript, since it is continuously referred to.

Detailed comments

The first movement part in a curl field disturbance may act as a contextual cue for the second, so maybe feedforward compensation and not feedback control is being learning during the experiment

In the abstract is states

"L8: A force field was applied during the first part of the movement and then unexpectedly switched off at the VP during catch trials"

You then say you use this paradigm in the current study and write

"L16 ... during the unexpected absence of the force field after VP, after-effects were observed"

In L53

"On catch trials, a force field was applied during the first part of the movement and then unexpectedly switched off after the via-point.”

Looking at figure 1 () which is nice and clear it seems to me that a more complete description in the first sentences above might be:

"L8: A force field was applied during the first and second parts of the movement and then occasionally unexpectedly switched off at the VP during catch trials"

Re-write this sentence to make it clearer: L30 Humans adapt to the change in environmental dynamics due to force fields that happens during reaching movements within a few minutes of practice

L35 The reasoning for the relative inability to learn opposing perturbations is that the motor memory attempts to learn the mean of the force fields applied on the recent trials

- Need to add given no contextual information here

Re-write this sentence to make it clearer: L44 However, more recent studies highlighted the possibility of concomitant learning of opposing and unexpected force fields expressed in the late phase of feedback correction that could be explained as the expression of online, continuous adaptive control

Ref twice? L46 (F. Crevecoeur et al., 2020; Frédéric Crevecoeur et al., 2020).

L66 Alternatively, if feedback-related changes in movement representation decay very fast, then trial-by-trial learning must be based on offline adjustments - Why not accumulated learning during trials?

L92 There were mainly seven types of movement conditions:

Mainly? It would also be helpful to illustrate all condition in the figure 1 as well

L107: So were the CW or CCW were randomly assigned on a trial-by-trial basis?

L111 : I think it’s better to say here: Via-point (VP) Baseline trials with slowdown at the via point"

L117 4 and 5

So, the dwell time does involve stopping but being slow and then the catch trials were conditioned on hand speed being < 3cms-1?

L123 Better to start “Once they reached the VP,....” In a new paragraph so the conditions are consistently presented.

L131 So participants performed an experiments with two different kind of blocks of type Either all the ShortDwell then all the LongDwell or vice versa?

L171 : Just seeing a reduction in MPE doesn’t just prove that online correction is occurring since there could be co-contraction.

More generally here the authours are already suggesting mechanisms in the results section here and its probably better to present the results first and then leave interpretation to later given all the evidence

183 Via-point dwell time: Maybe mention exactly how dwell time was calculated too here

200 Behavioral traces for movement through via-point

I found this section rather dense. It would, help to break it up a bit more into paragraphs

Also, it would help the readers if you focus more on the important aspects first and then mention the less important ones after.

Seems to me that the most interesting thing is looking at the aftereffects in the catch conditions for long dwell times compared to short dwell times

Also a vertical zero deviation line would be help in figure 4a and 4d

L229 ... there was no significant difference between ShortDwell and LongDwell trials for CCW

This seems like the most important result

L300 ... There was no significant difference between forcefield on/off situations in agonist and antagonist muscles.

This seems like the most important result from the EMG measurements

L337 We investigated how long do rapid changes in movement representation evoked by unexpected force field perturbations last within the course of a continuous sequence of movements.

Re-write this sentence to make it clearer

434 The effect of planning future movement was first shown by Howard et al 2015.

"The value of the follow-through derives from motor learning depending on future actions

IS Howard, DM Wolpert, DW Franklin - Current Biology, 2015"

A question the authors also raise is also is if there is an effect in two-part movements, then what are the implications for commonly used trial-by-trial learning paradigms.

L366 In trial-by-trial learning the inter-trial delays are typically several times more that 850ms, so I don’t see this can always explain what happens in trial-by-trial paradigms
